# Does guideline non-adherence result in worse clinical outcomes for hormone receptor-positive and HER2-negative metastatic breast cancer in premenopausal women?: result of an institution database from South Korea

**DOI:** 10.1186/s12885-018-5258-9

**Published:** 2019-01-17

**Authors:** Hee Kyung Kim, Soo-Hyeon Lee, Yu Jin Kim, Song Ee Park, Han Sang Lee, Sung Won Lim, Jang Ho Cho, Ji-Yeon Kim, Jin Seok Ahn, Young-Hyuck Im, Jong Han Yu, Yeon Hee Park

**Affiliations:** 1 Division of Hematology-Oncology, Departments of Internal Medicine, Samsung Medical Center, Sungkyunkwan University School of Medicine, 81 Irwon-ro, Gangnam-gu, Seoul, 06351 Korea; 2 Medical affairs Korea, Pfizer Oncology, Seoul, Republic of Korea; 3 Department of Internal Medicine, Chungbuk National University Hospital, Chungbuk National University College of Medicine, Cheongju, Republic of Korea; 4 Division of Breast Surgery, Department of Surgery, Samsung Medical Center, Sungkyunkwan University School of Medicine, 81 Irwon-ro, Gangnam-gu, Seoul, 06351 South Korea

**Keywords:** Metastatic breast cancer, Hormone receptor positive, Premenopausal, Chemotherapy

## Abstract

**Background:**

In this study, we observe the patterns initial palliative treatment for premenopausal patients with HR-positive/HER2-negative MBC and determine if nonadherence to clinical guidelines are associated with worse clinical outcomes in terms of progression-free survival (PFS) and overall survival (OS) in the South Korean population.

**Methods:**

A retrospective review was performed for premenopausal patients diagnosed with HR-positive/HER2-negative MBC between October 1997 and May 2016 who received palliative systemic treatments at a large tertiary medical center. Survival outcomes were analyzed according to the palliative treatment received prior to disease progression.

**Results:**

The review identified a total of 272 premenopausal patients meeting study criteria, whose median age was 39 years. Endocrine therapy was the initial treatment in 137 patients (Group 1) with chemotherapy as initial treatment in 135 patients. In the latter group, chemotherapy was continued in 78 patients (Group 2), whereas chemotherapy was switched to endocrine treatment in 57 patients prior to any disease progression (Group 3). Both PFS and OS were significantly longer for chemotherapy-endocrine therapy (median PFS 18.2 months and OS 85.2 months) than for chemotherapy-alone (median PFS 12.6 months and OS 45.5 months) or endocrine therapy-alone (median PFS 7.0 months and OS 57.3 months) (all *p* values < 0.01). In multivariate analysis, chemotherapy-endocrine therapy was an independent predictive value for improved PFS and OS (hazard ratio [HR] 0.33, 95% CI 0.20–0.52, *p* <  0.001; HR 0.38, 95% CI 0.19–0.73, *p* = 0.004).

**Conclusions:**

In our study population, chemotherapy alone was not objectively inferior to endocrine therapy as the initial palliative treatment. In addition, chemotherapy followed by endocrine therapy was associated with objective higher response rate than endocrine therapy alone. Further studies should explore the relationship between non-adherent treatment patterns and patient outcomes across the largely premenopausal breast cancer populations across Asian countries.

**Electronic supplementary material:**

The online version of this article (10.1186/s12885-018-5258-9) contains supplementary material, which is available to authorized users.

## Background

Breast cancer is the most common cancer in women worldwide and the leading cause of cancer death in women [1, 2]. Hormone receptor positive (HR-positive) subtype represents the majority of the patients with breast cancer (60–75%) [3], and early stage HR-positive patients receive adjuvant endocrine therapy after curative aim of treatment. Nevertheless, about 30% of early HR-positive breast cancer develops into metastatic disease over time, and de novo metastatic breast cancer represents about 5–10% of all breast cancer [4]. Despite the advancement in breast cancer management, metastatic breast cancer (MBC) continues to portend poor prognosis with 5-year survival rate of just 25% [2].

Endocrine therapy is the preferred option for the treatment of HR-positive, HER2-negative MBC, exclusive of visceral crisis or endocrine resistance [5]. So far, the consensus has been that initial palliative chemotherapy appears to be inferior to endocrine therapy in terms of efficacy and toxicity [6]. However, real-world practicing patterns differ from the guidelines with a considerable portion of patients with HR-positive/HER2-negative MBC still receiving initial palliative chemotherapy rather than endocrine therapy, with non-adherent practice resulting in worse outcomes [7].

In Asian populations, patients with breast cancer have distinct demographic characteristics compared to Western counterparts [8, 9]. The peak incidence for breast cancer is in the 40s among Asian patients, in contrast to the 60s in the United States [8]. The premenopausal patients make up about half of the whole breast cancer population in Asian countries, with approximately 10% of the patients being younger than 35 years [10, 11]. This must be understood in the context that breast cancer is known to be more aggressive and associated with poorer prognosis for premenopausal patients [12]. In this study, we observe the patterns of initial palliative treatment for premenopausal patients with HR-positive/HER2-negative MBC and determine if nonadherence to clinical guidelines are associated with worse clinical outcomes in terms of PFS and OS in the South Korean population.

## Methods

### Patients and data collection

Upon IRB approval, a retrospective review was performed for all patients who were treated for MBC at a tertiary cancer center between October 1997 and May 2016. For each patient with MBC diagnosis, the electronic medical record was reviewed for demographic information, Eastern cooperative oncology group (ECOG) performance status, HR status (expression of estrogen receptor (ER) and progesterone receptor (PgR)), HER2 expression, FSH level and menopausal status, and the type of treatment(s) received. Inclusion criteria were made for patients with premenopausal HR-positive/HER2-negative MBC who were treated with palliative aim, including chemotherapy and endocrine therapy. Premenopausal status was defined by last menstrual period within 12 months or FSH levels below 40 mIU/ml. Exclusion criteria consisted of follow-up loss before first evaluation of tumor response, double primary cancer with palliative chemotherapy, postmenopausal or unknown menopausal status, local recurrence followed by surgery and second adjuvant chemotherapy, positive for HER2 (confirmed by FISH or SISH), triple negative breast cancer, or unknown HR or unknown HER2 status.

### Statistical analysis

For statistical analysis, the patients were classified according to the first-line treatment received: chemotherapy, endocrine therapy, or chemotherapy-followed-by-endocrine therapy group. A patient was considered to have received both the chemotherapy and endocrine therapy as the first line treatment only if she was started on chemotherapy and switched to endocrine therapy without disease progression prior to discontinuing chemotherapy and beginning the endocrine therapy. Differences in baseline characteristics were examined using Pearson’s chi-square test and the one-way ANOVA test. Tumor response was assessed by the Response Evaluation Criteria in Solid Tumors (RECIST guideline, version 1.1). Progression-free survival (PFS) was measured from the date of first palliative therapy to the date of progression or date the patient was last seen. Overall survival (OS) was measured from the date of first palliative therapy to the date of death or date the patient was last seen. The Kaplan-Meier method was used for estimation of PFS and OS. Differences in survival were analyzed using the log-rank test, and a *p*-value less than 0.05 was considered significant. A multivariable Cox proportional hazard regression model was used to assess the impact of the prognostic variable on PFS and OS. Data were analyzed using the statistical software IBM SPSS 23.0 software (SPSS Inc., Chicago, IL, USA).

## Results

### Patients

**Fig. 1:**
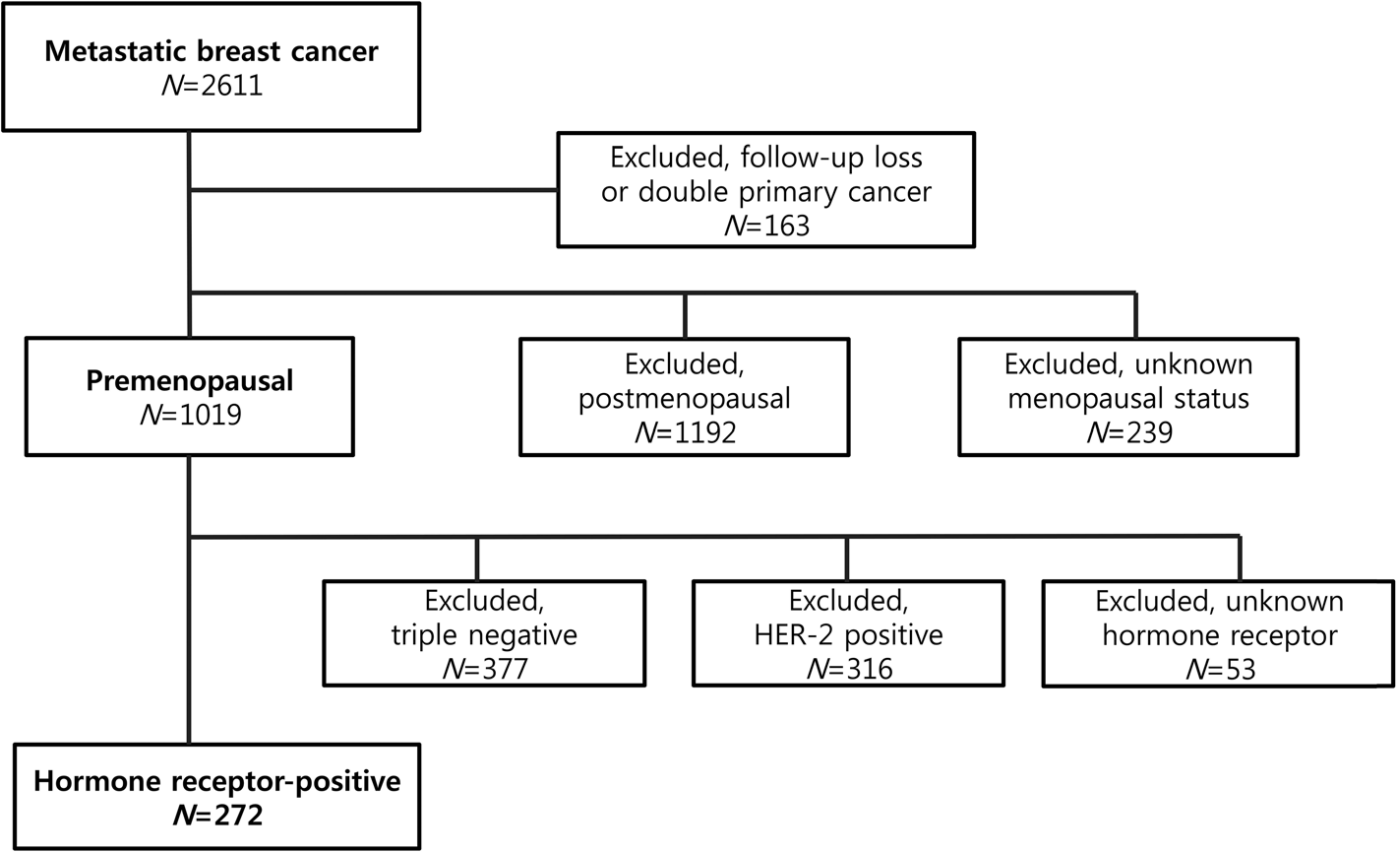
Consort diagram for hormone receptor-positive metastatic breast cancer. Of the total 2611 patients with MBC, confirmed premenopausal patients represented 39% (*N* = 1019). Ultimately, the review identified 272 premenopausal patients with HR-positive, HER2-negative MBC

The review of electronic patient database identified 2611 patients with MBC diagnosis who were treated with palliative therapy. Of these, 163 patients were excluded for follow-up loss or palliative therapy for double primary cancer. Further exclusions were made for postmenopausal patients (*N* = 1192) and patients of unknown menopausal status (*N* = 239). Among the remaining 1019 premenopausal patients with MBC, additional patients were excluded for triple negative (*N* = 377), HER2-positive (*N* = 316), and unknown HR status (*N* = 53) (Fig. 1).

In total, statistical analyses were performed for the remaining 272 patients with HR-positive/HER2-negative, premenopausal MBC. Palliative endocrine therapy was used as first-line palliative treatment in 137 patients (Group 1, 50.3%). Chemotherapy was used as first-line palliative treatment in 135 patients. Among these, the treatment was continued in 78 patients (Group 2, 28.7%), whereas chemotherapy was switched to endocrine treatment in 57 patients prior to any disease progression (Group 3, 21.0%) (Table 1). The therapy regimens are described in in the Additional file 1.

**Table 1:** Baseline characteristics

	Total (*N* = 272)	Endocrine therapy (*N* = 137)	Chemotherapy (*N* = 78)	Chemotherapy-endocrine therapy (*N* = 57)	*p* Value
Age, median (range)	39 (16–50)	40 (16–50)	37 (24–49)	39 (18–50)	0.111
ECOG (*N* = 191)					0.652
0	88 (46.1%)	43 (47.3%)	22 (38.6%)	23 (53.5%)	
1	100 (52.4%)	47 (51.6%)	34 (59.6%)	19 (44.2%)	
2	3 (1.6%)	1 (1.1%)	1 (1.8%)	1 (2.3%)	
Disease status					< 0.001
De novo	89 (32.7%)	25 (18.2%)	28 (35.9%)	36 (63.2%)	
Recurrent	183 (67.3%)	112 (81.8%)	50 (64.1%)	21 (36.8%)	
Disease-free interval in recurrent population (*n* = 176)					0.161
> 12 months between adjuvant Tx and recurrence	48 (27.3%)	30 (27.3%)	10 (20.8%)	8 (44.4%)	
≤ 12 months between adjuvant Tx and recurrence	128 (72.7%)	80 (72.7%)	38 (79.2%)	10 (55.6%)	
Disease site					< 0.001
Symptomatic visceral	63 (23.2%)	9 (6.6%)	34 (43.6%)	20 (35.1%)	
Asymptomatic visceral	69 (25.4%)	38 (27.7%)	19 (24.4%)	12 (21.1%)	
Bone and soft tissue only	140 (51.4%)	90 (65.7%)	25 (32.1%)	25 (43.9%)	
Receptor status					0.010
ER+ and PgR+	231 (84.9%)	116 (84.7%)	60 (76.9%)	55 (96.5%)	
ER+ and PgR-	34 (12.5%)	19 (13.9%)	13 (16.7%)	2 (3.5%)	
ER- and PgR+	7 (2.6%)	2 (1.5%)	5 (6.4%)	0	
FSH (*N* = 178)					0.989
median (range)	9.14 (0–40)	10.32 (0–39)	9.03 (1–38)	7.39 (2–40)	

*ECOG* Eastern Cooperative Oncology Group, *ER* estrogen receptor, *PgR* progesterone receptor, *Tx* treatment. All treatments were begun prior to disease progression

### Baseline patient characteristics

Baseline characteristics of the patients are depicted according to treatment groups in Table 1. As per the premenopausal inclusion criteria, the median age as 39 years (range 16–50) for the whole cohort. The majority of patients had ECOG PS of 0–1 (98.5%). Approximately two-third of cases represented recurrent disease after surgery with curative aim and adjuvant therapy (183/272, 67.3%) with the remaining patients having de novo stage IV breast cancer (32.7%). Among recurrent patients for whom follow up data was available (*N* = 176), the disease-free interval was less than 12 months (128/176, 72.7%), after completing 5 years of adjuvant endocrine treatment, which could deem this as a hormone resistant population. Overall, visceral metastasis was present in 48.6% of all patients; symptomatic visceral metastasis was present in 23.2%. De novo disease was more frequent for the chemotherapy-endocrine group (*p* <  0.001 and *p* = 0.010, respectively) Visceral metastasis was less frequent in the endocrine therapy group (*p* <  0.001).

### Treatment outcomes

**Fig. 2:**
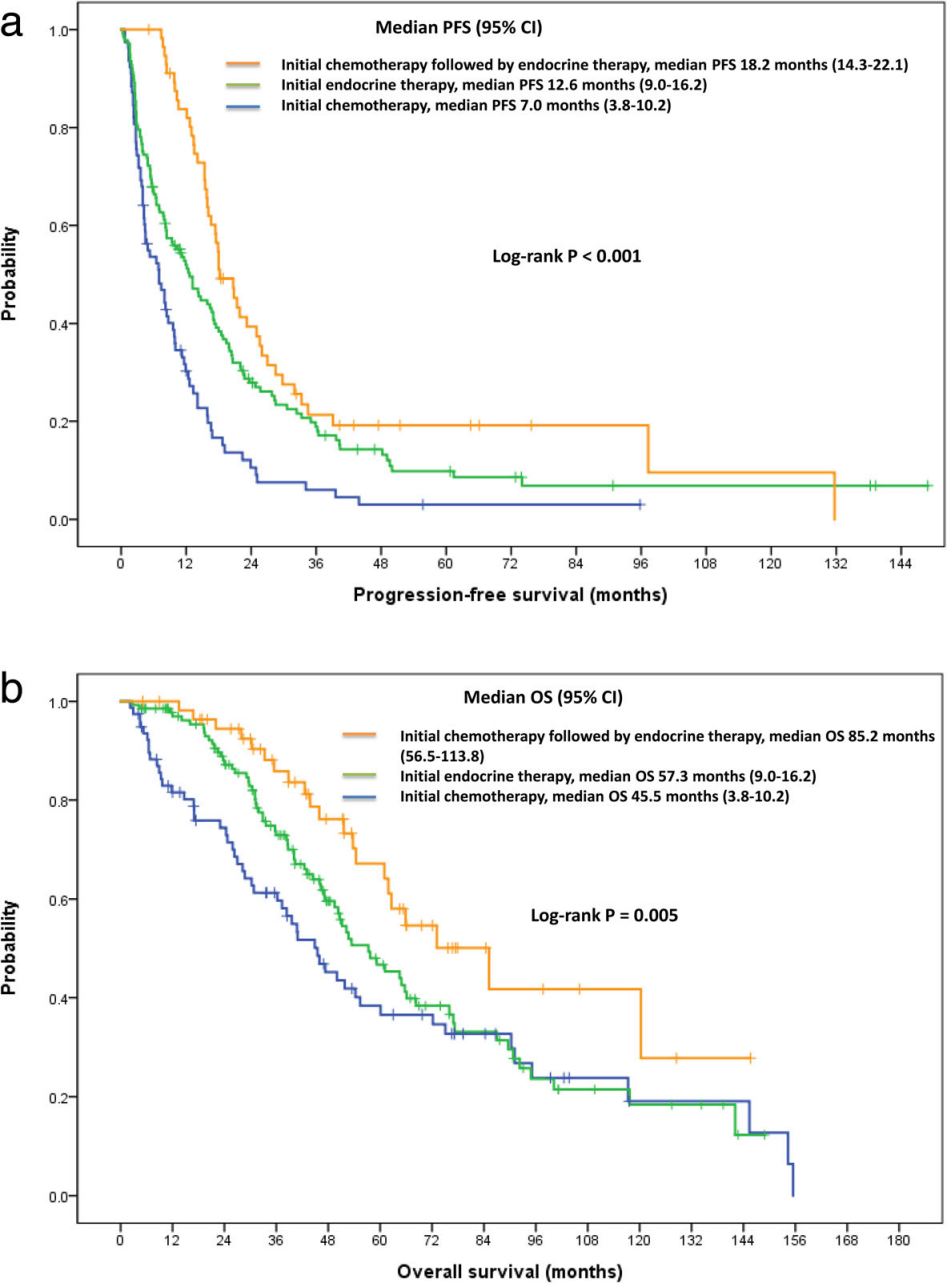
**a** and **b** Kaplan-Meier curves for progression-free survival (PFS) and overall survival (OS) according to the type of initial palliative treatment received. All treatments were considered to be initial palliative therapy only if begun prior to disease progression

For the overall cohort, the median follow-up period was 38.7 months (95% CI, 34.3–43.1). The median PFS for 1st line therapy and OS were 12.7 months (95% CI, 10.6–14.8) and 57.7 months (95% CI, 49.4–66.0), respectively. Treatment outcomes are demonstrated in Table 2 and Fig. 2. The PFS was significantly longer for the chemotherapy-endocrine therapy group (Group 3) than for the other two groups (Fig. 2a). The median PFS was 18.2 months (95% CI, 14.3–22.1) for chemotherapy-endocrine therapy group versus 12.6 months for endocrine therapy group (Group 1: 95% CI, 9.0–16.2) and 7.0 months for chemotherapy group (Group 2: 95% CI, 3.8–10.2), respectively (log-rank *p* <  0.001). Similar outcomes were observed for OS across the treatment groups. The median OS was 85.2 months (95% CI, 56.5–113.9) for Group 3, 57.3 months (95% CI, 45.6–69.0) for Group 1, and 45.5 months (95% CI, 34.9–56.1) for Group 2 with the differences being statistically significant (log-rank *p* = 0.005). Objective response rate (ORR) was significantly higher (80.7%) for Group 3 than for Groups 1 or 2 (26.9 and 26.3%, respectively; *p* = 0.011).

**Table 2:** Treatment outcome according to treatment strategy

	Initial endocrine therapy (*N* = 137)	Initial chemotherapy (*N* = 78)	Chemotherapy - endocrine therapy (*N* = 57)
Progression-free survival – mo			
Median	12.6	7.0	18.2
(95% CI)	(9.0–16.2)	(3.8–10.2)	(14.3–22.1)
Overall survival – mo			
Median	57.3	45.5	85.2
(95% CI)	(45.6–69.0)	(34.9–56.1)	(56.5–113.9)
Best response – no. (%)			
Complete response	7 (5.1%)	3 (3.8%)	7 (12.3%)
Partial response	29 (21.2%)	18 (23.1%)	39 (68.4%)
Stable disease	51 (37.2%)	24 (30.8%)	9 (15.8%)
Progressive disease	50 (36.5%)	33 (42.3%)	2 (3.5%)
Complete or partial response			
No. of patients (%)	36 (26.3%)	21 (26.9%)	46 (80.7%)
(95% CI)	(18.9–33.6)	(17.6–36.7)	(70.4–90.9)

*95% CI* 95% confidence interval. All treatments were begun prior to disease progression

Multivariate analysis identified the chemotherapy followed by endocrine therapy to be an independent prognostic factor of better outcome with a hazard ratio (HR) of 0.33 (95% CI, 0.20–0.52, *p* < 0.001) for PFS and 0.38 (95% CI, 0.19–0.73, *p* = 0.004) for OS. Whether followed by endocrine therapy or not, initial palliative chemotherapy was identified as a worse predictive factor for PFS (HR 1.72, 95% CI 1.17–2.52, *p* = 0.005), but this result did not extend to OS. Survival outcome did not correlate with other risk factors including younger age (≤ 35), poor performance (ECOG ≥1), or presence of visceral metastasis (Table 3).

**Table 3:** Multivariate Cox-regression model

	Progression-free survival (PFS)		Overall survival (OS)	
	HR (95% CI)	*p* Value	HR (95% CI)	*p* Value
Age ≤ 35	1.16 (0.81–1.66)	0.431	1.21 (0.76–1.93)	0.425
ECOG ≥1	1.55 (1.10–2.16)	0.011	1.51 (0.96–2.36)	0.074
Visceral metastasis	1.27 (0.91–1.78)	0.156	1.25 (0.79–2.00)	0.344
PgR-negative	1.61 (0.99–2.62)	0.056	1.56 (0.85–2.85)	0.152
Initial endocrine therapy	0.59 (0.41–0.88)	0.009	0.73 (0.43–1.22)	0.224
Chemotherapy-endocrine therapy	0.34 (0.21–0.55)	< 0.001	0.40 (0.20–0.78)	0.007

*HR* hazard ratio, *95% CI* 95% confidence interval, *ECOG* Eastern Cooperative Oncology Group, *PgR* progesterone receptor. All treatments were begun prior to disease progression

## Discussion

Our current study was conceived from the discrepancy between evidence-based guidelines and the real-world practice pattern for MBC patients in South Korea. While we understand that the major guidelines recommend endocrine therapy as the preferred and standard treatment for HR-positive/HER2-negative MBC [5, 13], our experience has been that chemotherapy is frequently used as the first line treatment in a significant proportion of these patients without visceral crisis. For the past decade, we have suspected that this discrepancy between the guidelines and real-world practice was due to the fact that a larger proportion of our MBC patients are premenopausal - a suspicion that now finds support in this study.

To review the relevant literature, the pivotal studies on the efficacy of endocrine therapy vs. chemotherapy were conducted mostly in postmenopausal women (median age between 65 and 66) [14–16], with more recent trials conducted for patient in the 7th decade of life (median age between 61 and 62) [17, 18]. For the management of premenopausal MBC patients, guidelines recommend endocrine therapy with ovarian ablation or suppression and to follow through with postmenopausal treatment guideline [19]. However, ovarian ablation or suppression has not been assessed in efficacy versus chemotherapy in the younger MBC population, and chemotherapy continues to be administered to a large portion of HR-positive/HER2-negative MBC patients, including premenopausal women [7, 20].

The discrepancy between treatment guideline and real-world practice pattern has most recently been evaluated by the Southern Netherlands Breast Cancer Consortium [7]. The study evaluated treatment patterns for 482 patients with HR-positive/HER-2 negative MBC and have found that one-quarter of patients (116, 24%) have received chemotherapy as initial palliative therapy (against the current guideline recommendation), whereas endocrine therapy as initial palliation was given in three-quarters of patients (366, 76%). The two main take-away finding from the study was that patients who received chemotherapy as initial palliation were more likely to be younger (median age 52 vs 61 years, *p* < 0.0001) and experienced worse outcomes in terms of PFS (HR 2.33, *p* < 0.0001) and OS (HR 2.24, *p* < 0.0001). These findings suggest that the failure to comply with the guideline was associated with worse outcomes in the Dutch population, despite the fact that patients receiving chemotherapy were about a decade younger and had significantly lower frequency of comorbidity than patients receiving endocrine therapy.

In our study, chemotherapy alone as initial palliative treatment was not associated with the conclusive difference in outcomes, as that would have been consistent with the Dutch study. While initial endocrine therapy was associated with improved progression-free survival over initial chemotherapy, the overall survival was not statistically different between the two groups. Moreover, the group of patients receiving chemotherapy followed by endocrine therapy experienced significantly improved outcomes compared to either chemotherapy or endocrine therapy alone. Here, we pointed-out that chemotherapy followed by endocrine maintenance therapy is not intended from the beginning. We did chemotherapy because the patients were not appropriate for endocrine therapy for several reasons at diagnosis of metastasis; higher tumor burden, symptomatic visceral metastases, and aggressive tumor behavior, and etc. However, after showing chemotherapy response, they could receive endocrine therapy maintenance till progression. There may be a clinically distinct group among ER-positive populations who could have benefit from chemotherapy followed by maintenance endocrine therapy especially for premenopausal population. A recent Korean multi-omics study showed a plausible explanation that Korean BC was independently associated with increased tumor-infiltrating lymphocyte (TIL) and decreased transforming growth factor (TGF)-signaling expression signatures, suggesting that Asian tumors may harbor a different biology [21]. Overall, our study result stands in stark contrast from that of the Dutch study and raises the question of why chemotherapy appears to be just as appropriate as the initial palliative treatment choice as endocrine therapy in our study population.

At a glance, our single-institutional result can easily be dismissed as an isolated observation, which is a valid yet refutable criticism. Our institution is one of the largest tertiary care centers within S. Korea that takes care of 10% of the national breast cancer population [9]. More so, South Korea is not alone in the demographic change to continuing rise of chronic disease as the cause of death [21]. It has only been within the past 10 years that we have begun to realize breast cancer presents 20 years earlier in Asian populations than in Western populations [8]. From both epidemiologic and biochemical perspectives, the studies seem to suggest that HR-positive/HER2-negative MBC in our patients might represent a distinct clinical entity than that reported for patients in Western countries.

Our study does have some significant weaknesses. As per the retrospective study design, we can only build associations between the treatment patterns and outcomes. It would be just as easy to argue that the excellent outcome for the chemotherapy-endocrine group represents a cherry-picked evidence, though the chemotherapy alone itself was not significantly objective worse in outcomes than endocrine therapy alone. Possible confounding factors associated with treatment choices include comorbidity and patient preference. Another significant difference may be that novel agents such as CDK 4/6 inhibitors, fulvestrant, or everolimus are not routinely prescribed in S. Korea because the national health insurance program does not cover the cost of these drugs.

In conclusion, our study renders a different perspective to initial palliative chemotherapy for HR-positive/HER2-negative MBC in the premenopausal Asian population. In the study population, chemotherapy alone was not objectively inferior to endocrine therapy as the initial palliative treatment. In addition, chemotherapy followed by endocrine therapy was associated with objectively higher response rate than endocrine therapy alone. Our observation does not agree with the Dutch study finding of worse outcome for guideline nonadherence. The working hypothesis for the difference between the Dutch study and ours is that our population is distinct from the patient populations for which the guidelines have developed. Further prospective studies should explore the relationship between non-adherent treatment patterns and patient outcomes across the largely premenopausal breast cancer populations across Asian countries.

## Conclusions

Chemotherapy alone was not objectively inferior to endocrine therapy as the initial palliative treatment for premenopausal patients with HR-positive/HER2-negative MBC.

## Additional file

Additional file 1: **Supplementary Methods.** Treatment – endocrine therapy. Treatment – chemotherapy. Treatment – chemotherapy followed by endocrine therapy. **Table S1.** Frequency of endocrine therapy. **Table S2.** Frequency of chemotherapy agent. **Table S3.** Frequency of chemotherapy followed by endocrine therapy. (DOCX 30 kb)

## Abbreviations

ECOG: Eastern cooperative oncology group
ER: Estrogen receptor
HR: Hazard ratio
HR-positive: Hormone receptor positive
MBC: Metastatic breast cancer
ORR: Objective response rate
OS: Overall survival
PFS: Progression-free survival
PgR: Progesterone receptor
RECIST: Response evaluation criteria in solid tumors
