# Supplementary material for: Does guideline non-adherence result in worse clinical outcomes for hormone receptor-positive and HER2-negative metastatic breast cancer in premenopausal women?: result of an institution database from South Korea
Source: BMC Cancer. 2019 Jan 17;19:84. doi: 10.1186/s12885-018-5258-9 (PMC6337867; doi:10.1186/s12885-018-5258-9)
Supplement: Supplementary file 1 — Supplementary Methods. Treatment – endocrine therapy. Treatment – chemotherapy. Treatment – chemotherapy followed by endocrine therapy. Table S1. Frequency of endocrine therapy. Table S2. Frequency of chemotherapy agent. Table S3. Frequency of chemotherapy followed by endocrine therapy. (DOCX 30 kb) [file 12885_2018_5258_MOESM1_ESM.docx]

**Additional file 1**

**Supplementary Methods**

***Treatment – endocrine therapy***

(1) Tamoxifen: oral tamoxifen 20 mg daily

(2) Letrozole: oral letrozole 2.5 mg daily

(3) Anastrozole: oral anastrozole 1 mg daily

(4) Exemestane: oral exemestane 25 mg daily

(5) Gosereline: Goserelin 3.6 mg subcutaneously every 4 weeks

(6) Tamoxifen with goserelin: oral tamoxifen 20 mg daily + gosereline depot 3.6 mg subcutaneously every 4 weeks

(7) Letrozole with goserelin: oral letrozole 2.5 mg daily + gosereline depot 3.6 mg subcutaneously every 4 weeks

(8) Anastrozole with goserelin: oral anastrozole 1 mg daily + gosereline depot 3.6 mg subcutaneously every 4 weeks

(9) Others

- Palbociclib with fulvestrant (PALOMA-3 study): D1-D21 Palbociclib 125 mg or placebo daily + D1 fulvestrant 500 mg intramuscular injection every 4 weeks

- Buparisib with tamoxifen and gosereslin (B-YOND study): D1 Goserelin 3.6 mg subcutaneously + D1-D28 oral Buparlisib 100 mg with tamoxifen 20 mg daily every 4 weeks

- Ribociclib/placebo with letrozole and goserelin (MONALEESA-7 study): D1 Goserelin 3.6 mg subcutaneously + D1-D21 oral ribociclib 600 mg daily/placebo + D1-D28 tamoxifen 20 mg daily every 4 weeks

- Taselisib with fulvestrant (SANDPIPER study): D1-D28 oral Taselisib 4 mg daily + D1, D15 fulvestrant 500 mg intramuscular injection every 4 weeks

- Abemaciclib with fulvestrant (MONARCH 1 study): D1-D28 oral Abemaciclib 200 mg daily + D1, D15 fulvestrant 500 mg intramuscular injection every 4 weeks

- Toremifene: oral toremifene 60 mg daily every 4 weeks

***Treatment - chemotherapy***

(1) AC: Cyclophosphamide 600 mg/m^2^ + doxorubicin 60 mg/m^2^ every 3 weeks intravenously (IV)

(2) Paclitaxel monotherapy: Paclitaxel 175 mg/m^2^ every 3 weeks IV
(One patient received weekly paclitaxel : D1, D8, D15 paclitaxel 80 mg//m^2^ every 3 weeks IV)

(3) Paclitaxel with cisplatin: Paclitaxel 175 mg/m^2^ + cisplatin 60 mg/m^2^ every 3 weeks IV

(4) Paclitaxel with carboplatin: Paclitaxel 150 mg/m^2^ + carboplatin AUC 5.5 every 3 weeks IV

(5) Paclitaxel with gemcitabine: D1 Paclitaxel 175 mg/m^2^ + D1, D8 gemcitabine 1250 mg/m^2^ every 3 weeks IV

(6) Docetaxel monotherapy: Docetaxel 75 mg/m^2^ every 3 weeks IV

(7) Docetaxel with capecitabine: D1 docetaxel 75 mg/m^2^ + D1-D14 oral capecitabine 1250 mg/m^2^ every 3 weeks

(8) Capecitabine monotherapy: D1-D14 oral capecitabine 1250 mg/m^2^ every 3 weeks

(9) Vinorelbine with capeciabine: D1, D8 vinorelbine 25 mg/m^2^ IV + D1-D14 oral capecitabine 1250 mg/m^2^ every 3 weeks

(10) Eribulin with gemcitabine: D1, D8 Eribulin 1.0 mg/m^2^ + D1, D8 gemcitabine 1000 mg/m^2^ every 3 weeks IV

(11) Others
- Docetaxel with ramucirumab (TRIO-012 study): Docetaxel 75 mg/m^2^ + ramucirumab 10 mg/kg every 3weeks IV
- Paclitaxel with lapatinib (EGF30001 study)
- AT: Docetaxel 75 mg/m + doxorubicin 60 mg/m^2^ every 3 weeks

- Docetaxel with bevacizumab (AVADO study): Docetaxel 75 mg/m^2^ + bevacizumab 7.5 mg/kg every 3 weeks IV

- 5-FU with medroxyprogesterone (regimen unknown – outside hospital)

***Treatment – chemotherapy followed by endocrine therapy***

(1) AC: Cyclophosphamide 600 mg/m^2^ + doxorubicin 60 mg/m^2^ every 3 weeks intravenously (IV); followed by tamoxifen: oral tamoxifen 20 mg daily

Or followed by tamoxifen with goserelin: oral tamoxifen 20 mg daily + gosereline depot 3.6 mg subcutaneously every 4 weeks

Or followed by anastrozole with goserelin: oral anastrozole 1 mg daily + gosereline depot 3.6 mg subcutaneously every 4 weeks

(2) Paclitaxel with carboplatin: Paclitaxel 150 mg/m^2^ + carboplatin AUC 5.5 every 3 weeks IV; followed by gosereline: Goserelin 3.6 mg subcutaneously every 4 weeks

(3) Weekly paclitaxel: D1, D8, D15 paclitaxel 80 mg//m^2^ every 3 weeks IV;
followed by tamoxifen: oral tamoxifen 20 mg daily

(4) Docetaxel with cisplatin: Docetaxel 75 mg/m^2^ + cisplatin 60 mg/m^2^ every 3 weeks IV;
followed by gosereline: Goserelin 3.6 mg subcutaneously every 4 weeks

(5) Docetaxel monotherapy: Docetaxel 75 mg/m^2^ every 3 weeks IV;
followed by tamoxifen: oral tamoxifen 20 mg daily

*All endocrine therapy was begun prior to disease progression*.*

**Table S1** Frequency of endocrine therapy

|  | Endocrine therapy group  (*n* = 137) |
| --- | --- |
| Tamoxifen | 41 (29.9%) |
| Letrozole | 27 (19.7%) |
| Anastrozole | 3 (2.2%) |
| Exemestane | 1 (0.7%) |
| Gosereline | 24 (17.5%) |
| Tamoxifen with gosereline | 13 (9.5%) |
| Letrozole with gosereline | 16 (11.7%) |
| Anastrozole with gosereline | 4 (2.9%) |
| Others | 8 (5.8%) |

**Table S2** Frequency of chemotherapy agent

|  | Chemotherapy group  (*n* = 78) |
| --- | --- |
| AC | 20 (25.6%) |
| Paclitaxel monotherapy | 6 (7.7%) |
| Paclitaxel with cisplatin | 2 (2.6%) |
| Paclitaxel with carboplatin | 2 (2.6%) |
| Paclitaxel with gemcitabine | 14 (17.9%) |
| Docetaxel monotherapy | 7 (9.0%) |
| Docetaxel with capecitabine | 7 (9.0%) |
| Capecitabine monotherapy | 8 (10.3%) |
| Vinorelbine with capeciabine | 1 (1.3%) |
| Eribulin with gemcitabine | 4 (5.1%) |
| Others | 7 (9.0%) |

**Table S3.** Frequency of chemotherapy followed by endocrine therapy

| Initial chemotherapy (*n* = 57) | | Endocrine therapy (*n* = 57)* | |
| --- | --- | --- | --- |
| AC | 48 (84.2%) | Tamoxifen | 41 (71.9%) |
| Paclitaxel monotherapy | 2 (3.6%) | Letrozole | 4 (7.0%) |
| Paclitaxel with carboplatin | 3 (5.3%) | Anastrozole | 1 (1.8%) |
| Docetaxel monotherapy | 3 (5.3%) | Gosereline | 5 (8.8%) |
| Docetaxel with cisplatin | 1 (1.8%) | Tamoxifen with gosereline | 5 (8.8%) |

*After initial chemotherapy, endocrine therapy was begun prior to disease progression.
